# Iodoform as a Dressing, Especially in Tubercular Ulcerations of Bones and Joints

**Published:** 1881-07

**Authors:** Frederick Peterson

**Affiliations:** Vienna


### IODOFORM AS A DRESSING, ESPECIALLY IN TUBERCULAR ULCERATIONS OF BONES AND JOINTS.—BY MIKULICZ (VIENNA.)

FROM THE GERMAN BY FREDERICK PETERSON, M. D.

SINCE New Year, 1881, iodoform has been used in various ways at Billroth's clinic. Besides tubercular-fungous ulcerations it was used in fresh, gangrenous and diphtheritic sores, not amenable to antiseptic treatment, and with most excellent results. Especially was such success attained in its application to fresh ulcerations in the mouth, pharynx and rectum, beyond that of any other remedy, that according to Mikulicz it must become one of the most important means in the cure of wounds.

According to Binz, Högyes, Moleschott and Oberländer, the local action of iodoform is to be considered as the protracted action of iodine. Through the continual decomposition of the iodoform the iodine works powerfully *in statu nascendi*, without the irritation of tissue, which results from the use of tinct. iodine. Although iodine may continually be discovered in the urine, no serious symptoms of poisoning from its use occur. The writer has studied the antiseptic properties of iodoform. Various organic fluids, as Pasteur's solutions, one per cent. beef extract, one per cent. malt extract, one per cent. pepton solution, beef water, blood, and urine were used, some being treated with large quantities of iodoform and others let alone. In some of the liquids very small quantities of iodoform were used, but it was found that it wasted rapidly by volatilization, unless employed in greater quantities. The solutions were set in an oven to putrefy. In all, the iodoform disclosed antiseptic and anti-bacterial properties; weak where small quantities were used, but when larger were introduced, prompt to destroy all bacterial development and tendency to disintegration. In thin solutions of blood the development of fungi was only retarded, not wholly prevented, but putrefaction failed to take place.

Iodoform is used in the form of powder; in a stick with one part iodoform to two of gelatine, in emulsion; in an ether solution, one part to five.

Rules for application: The remedy is brought directly in contact with the wound, covered with iodoform gauze, and over that wadding and a moistened cloth are placed. A change is to be made in two to four days, and after that every week or two.

Besides a multitude of small cases, thirty-six severe ones were in this way treated, as follows:

Eight fresh wounds (great sarcoma of the omentum, carcinoma recti, extirpation of larynx for carcinoma, angioma linguæ et buccæ, etc.) Five ulcers and diphtheritic wounds (phagedenic ulcer of the hand, in which iodoform exhibited a remarkable anodyne action; necrosis of the index finger, etc.) Twenty-three fungo-tubercular ulcers (extensive caries of the knee-joint with destruction of the epiphysis of the femur and numerous fistulæ healed and consolidated by iodoform in two and a half months; caries of elbow-joint healed in one and a half months; caries of hip-joint with numerous fistulæ; extensive tubercular infiltration of the soft parts of the arm and forearm, etc.) Further, in a case of fungous of the ankle-joint without fistulæ parenchymatose injections of ether solution of iodoform were made, and after their use fifteen times the mass died away. In a case of lupus of the foot a clean granulating surface with cicatrization was secured by simply sprinkling with iodoform.

Herr Mikulicz arrives at the following conclusions: 1. Iodoform is an excellent antiseptic for all sorts of sores, but only by direct contact. In sores so treated there is no local irritation, the secretion is at its minimum and more serous; there is no fever, no pain; bandages are changed seldom and simply applied. 2. In the form of iodoform gauze pads it excels for simplicity and safety in sores of the mouth, pharynx, alimentary canal, vagina, etc., all remedies heretofore applied for aseptic and anodyne purposes. 3. It is an excellent cleanser of ulcers and sloughing sores. 4. It has a specific influence upon fungo-tubercular granulation, but only by direct application. They change to clean, fresh, healthy granulations which soon cicatrize.

A distant anti-tubercular action in other forms of the disease or a general anti-tubercular action has not been established.

Discussion: Gussenbauer, of Prague, having used iodoform since January 1, also, in 19 severe cases of joint disease, comes to the following almost similar conclusion: 1. A perfect aseptic healing of the sores is guaranteed. 2. The tendency of granulations to become fungous, is prevented, and they are led to form a dense and solid cicatricial tissue. Seven cases of resection were already healed; although he often used large doses of iodoform—in one case as high as 200 grains in substance—he had never met with a threatening toxic symptom.—*Supplement of Centralblatt fuer Chirurgie.*

